# Ultrasound-Guided High-Intensity Focused Ultrasound of Uterine Fibroids and Adenomyosis: An 11-Year Experience from a Single Center in Hong Kong

**DOI:** 10.3390/jcm13164788

**Published:** 2024-08-14

**Authors:** Vivian Wai-Yan Ng, Vincent Yuk-Tong Cheung

**Affiliations:** Department of Obstetrics and Gynaecology, Queen Mary Hospital, University of Hong Kong, Hong Kong, China

**Keywords:** uterine fibroids, adenomyosis, ultrasound-guided high-intensity focused ultrasound (HIFU), high-intensity focused ultrasound ablation, quality of life

## Abstract

**Introduction:** This study evaluated the efficacy and safety of ultrasound-guided high-intensity focused ultrasound (HIFU) in treating symptomatic uterine fibroids and adenomyosis. **Methods:** HIFU treatments performed in premenopausal women with symptomatic uterine fibroids and adenomyosis were analyzed retrospectively. Lesion volume reduction, change in symptoms of menstrual pain, and quality of life were examined. Major and minor complications, together with re-intervention rates, were evaluated. **Results:** Eighty-one HIFU treatments were performed in seventy-nine premenopausal women. The follow-up period was up to 95 months. A total of 65 women underwent treatment for uterine fibroids and 14 were treated for adenomyosis. For patients with uterine fibroids, the baseline fibroid volume median was 190.1 cm^3^ (18.5–1729.4 cm^3^). Fibroid volume was reduced by 50.1% (−26.2–97.8, *p* < 0.0001) at 6 months and 66.9% (−33.7–98.3, *p* < 0.0001) at 12 months after treatment. The modified Uterine Fibroid Symptom and Quality of Life (UFS-QOL) scores had decreased by 43.5% (0–62.5%, *p* < 0.0001) at 6 months and 50% (0–73%, *p* < 0.0001) at 12 months after treatment. In the adenomyosis arm, the median baseline uterine volume was 97.7 cm^3^ (43.7–367.4 m3). Uterine volume was reduced by 19.6% (range: 1.2–42.0, *p* = 0.28) at 6 months and 41.9% (18.9–69.2, *p* = 0.04) at 12 months after treatment. UFS-QOL scores were reduced by 38.1% (6–66.7%, *p* < 0.0001) at 6 months and 40% (0–70%, *p* < 0.0001) at 12s month after treatment. Fourteen (21.5%) patients with uterine fibroid and five (35.7%) patients with adenomyosis required subsequent interventions. **Conclusions:** HIFU provides symptomatic relief to most patients with uterine fibroids and adenomyosis. It is a promising uterus-sparing treatment for patients with these conditions.

## 1. Introduction

Uterine fibroids and adenomyosis are two of the most common uterine benign lesions and cause debilitating symptoms such as abnormal menstrual bleeding and pelvic pain. The lifetime risk is up to 60% in women aged over 45 years [1]. For many years, the gold standard has been surgical management when a patient fails medical therapy. However, this carries significant operative risks and could affect fertility potential. Many minimally invasive treatment options are now available, including uterine artery embolization, radiofrequency ablation, and high-intensity focused ultrasound (HIFU) [2]. While no large studies compare the efficacy of HIFU with pharmaceutical methods, we generally use medical therapies as the first-line therapeutic option.

HIFU emerges as a uterus-sparing option for women who seek treatment alternatives for uterine fibroid and adenomyosis. Focal thermocoagulation of the target lesion induces lesion shrinkage. In the past, magnetic resonance imaging (MRI) has been used to monitor ablation [3,4,5]. Ultrasound-guided HIFU (HIFU) has also been proven to be safe and effective in reducing the lesion volume while achieving significant symptom relief [6,7,8].

The JC HIFU system was installed in 2006 at Queen Mary Hospital for treatment of hepatocellular carcinoma [9], and in 2012, treatment of uterine fibroid was started. Our preliminary experience on the application of HIFU on the first 20 patients has demonstrated a significant fibroid volume reduction of 75.9% and improvement in the symptom severity score by 44.9% at 12 months after HIFU [10]. Since 2016, our service has expanded to include patients with adenomyosis, and in 2023, we published the outcomes of the treatment for the first ten patients [11].

In Hong Kong, Queen Mary Hospital has pioneered the development of HIFU in the treatment of fibroid and adenomyosis [12], and at present, it is the only center under public healthcare that provides this treatment modality. We trust that our review can provide useful data with which to facilitate the counseling of women who wish to consider this treatment. The objective of this study is to review the therapeutic efficacy, safety, and long-term outcomes of all patients who received HIFU treatment for uterine fibroids and adenomyosis since the introduction of this treatment modality in our center in 2012.

## 2. Materials and Methods

**Figure 1:**
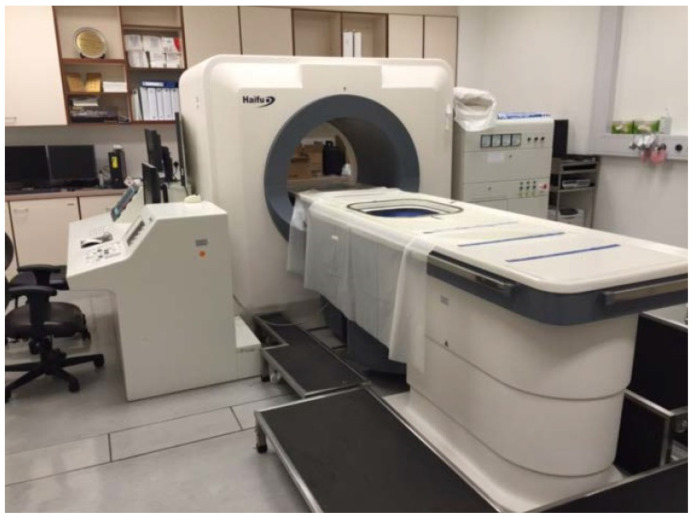
Ultrasound-guided high-intensity focused ultrasound system.

In our center, the HIFU system (JC model, Chongqing Haifu Technology Company, Chongqing, China) was used (Figure 1). All patients had pre-treatment MRI for assessment and planning. Patients were considered eligible for HIFU if the following criteria were met: (1) pre-menopausal women over 35 years of age, with special consideration for women less than 35; (2) symptoms related to the dominant uterine fibroid or adenomyosis intractable to standard medical therapy; (3) abdominal wall thickness < 5 cm; (4) uterus size < 24 weeks gestation on clinical examination; and (5) dominant uterine fibroid diameter < 12 cm or localized adenomyotic lesion < 10 cm via MRI. Women with a history of abdominal or pelvic surgery resulting in extensive scarring of the lower abdominal wall, especially those with midline and/or repeated laparotomy scars, were excluded. Previous reports from our center have outlined the details of HIFU therapy [10,11]. Each patient had pre-treatment planning prior to treatment to simulate the course of therapy, which involved an evaluation of the sonication path, target depth, distance from sacrum to target, and chance of bowel loop existence along the path. All patients had mechanical bowel preparation via phospho-soda fleet prior to HIFU therapy. A urinary catheter was placed in the bladder to control the bladder volume. Patients were placed in a prone position under intravenous conscious sedation. The target uterine lesion was localized using ultrasound. With the therapeutic transducer’s acoustic power output at 350–400 W and continuous sweeps from the deep to shallow region, the desired volume of the target fibroid or adenomyoma were ablated using real-time ultrasound monitoring. The grey scale change was observed with ultrasound and used to determine the ablation adequacy. If necessary, post-treatment analgesics, such as paracetamol and diclofenac, were used to relieve discomfort. After twenty-four hours, patients were discharged.

All patients had MRI at 6 months and ultrasound at 12 months after treatment to measure the volume (V) of the fibroids, adenomyotic lesions, and for patients with adenomyosis, the uterus, using the following formula: V = 0.5233 × D1 × D2 × D3, where D1, D2, and D3 indicated the dimension in longitudinal, anteroposterior, and transverse planes. All patients had completed an eight-item section of a Uterine Fibroid Symptom and Quality of Life Questionnaire (UFS-QOL) at baseline, as well as during follow-up at 6- and 12-months post treatment. This symptoms severity score uses eight questions, assessed on a 5-point scale, to assess both bleeding and pressure symptoms. Responses are scored from 1 (not at all) to 5 (a very great deal), with possible results from 8 to 40. Patients with adenomyosis had also completed the menstrual pain score, which assessed the degree of menstrual pain using a 10-point scale from 1 (not at all) to 10 (a very great deal). Complications were categorized based on the standards as defined by the Society of Interventional Radiology (SIR) Standards of Practice Committee Classification of Complications by Outcome [13]. Major complications were defined as those requiring therapy or minor hospitalization of less than 48 h (Class C); requiring major therapy, unplanned increase in the level of care, or prolonged hospitalization of more than 48 h (Class D); having permanent adverse sequelae (Class E); or resulting in death (Class F) [13].

This was a retrospective review which collected clinical data from all patients who underwent HIFU treatment for uterine fibroid and adenomyosis at Queen Mary Hospital between March 2012 and July 2023. Background information, pre-treatment lesion characteristics, treatment details, and clinical outcomes were retrieved. Data were expressed as median and range, and the Wilcoxon signed rank test was used for comparison between changes in outcome measures. *p* < 0.05 was considered statistically significant. Statistical analysis was conducted using SPSS version 25.0 statistical software. This study was approved by the Hospital Authority Hong Kong West Cluster Institutional Review Board (Ref No.: UW 20-619). Informed consent was waived as this is a retrospective study.

## 3. Results

A total of 81 HIFU treatments were performed in 79 premenopausal women. As many as 65 women underwent treatment for 75 fibroids. One woman with three fibroids received two treatments via a two-stage procedure. Fourteen women received treatment for adenomyosis. Table 1 summarizes the patient characteristics and treatment details. All patients were symptomatic with menorrhagia, dysmenorrhea, or pressure-related symptoms due to an enlarged uterus. Two patients with fibroids had significant premorbid medical conditions, including one with Eisenmenger syndrome and another one with deep vein thrombosis.

### 3.1. Fibroids

**Figure 2:**
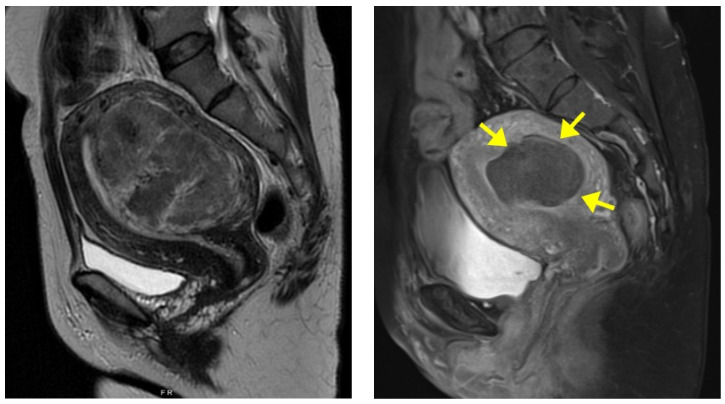
Magnetic resonance (MR) images of a fibroid from a 40-year-old woman; (**Left**) pre-HIFU MR image showing a large anterior uterine fibroid; (**Right**) Post-HIFU MR image, 6 months after treatment, showing a hypoperfused well-defined area (arrows) as the result of HIFU with a 73.9% reduction in fibroid volume.

Of the 75 fibroid cases, 28 (37.3%) were Type 2 fibroids according to the International Federation of Gynaecology and Obstetrics (FIGO) classification [14], 17 (22.7%) were Types 2–5, 13 (17.3%) were Types 3–4, 13 (17.3%) were Type 5, and 4 (5.4%) were Type 6. The baseline fibroid volume median was 190.1 cm^3^ (range: 18.5–1729.4 cm^3^). Table 2 summarizes the fibroid volumes at 6 and 12 months after treatment and their corresponding percentage volume reduction. The images of one of the patients before and 6 months after HIFU are shown in Figure 2. The symptom severity scores (SSS) using the modified UFS-QOL questionnaire median before treatment was 28 (range: 19–35). The scores at 6 and 12 months after treatment are summarized in Table 2.

The longest duration of follow-up was 95 months. Thirty-eight (58.5%) patients had attended follow-up for more than 2 years. A total of 27 women (41.5%) had completed follow-up or had been referred out to primary care, 12 (18.5%) women had defaulted or left the country after receiving treatment, and 26 women (40.0%) are still attending ongoing follow-ups. Fourteen patients (21.5%) required additional intervention. Five patients had hysterectomy 20 to 52 months after HIFU due to persistent heavy menstrual bleeding, except one patient, who had MR imaging suspicious of sarcoma, which was not confirmed on final histology. A total of 7 patients (10.8%) had vaginal and/or hysteroscopic myomectomy 10 to 80 months after HIFU, and 2 patients (3.1%) had levonorgestrel-releasing intrauterine system insertion at 18 months. Although the overall re-intervention rate came to 21.5%, if one considers HIFU as a treatment with which to facilitate subsequent hysteroscopic myomectomy for uterine preservation, the adjusted re-intervention rate was only 10.8%.

Most complications were minor (Table 1). One patient (1.5%) had pelvic inflammatory disease and E coli septicemia 3 weeks after HIFU, which resolved after antibiotics therapy but required hospitalization for 7 days (SIR Class D). Two patients (3.1%) had second-degree skin burn, with one occurring over the previous tubal ligation scar. Both were classified as SIR Class B and resolved without additional treatment. Two patients (3.1%) had urinary tract infection requiring antibiotics therapy (SIR Class B). The most common adverse event after treatment was mild post-treatment pelvic, leg, and back pain in eight patients (12.3%); the pain lasted less than one week in five patients and less than three months in three patients. Eight women had reached menopause during their follow-up duration.

### 3.2. Adenomyosis

**Figure 3:**
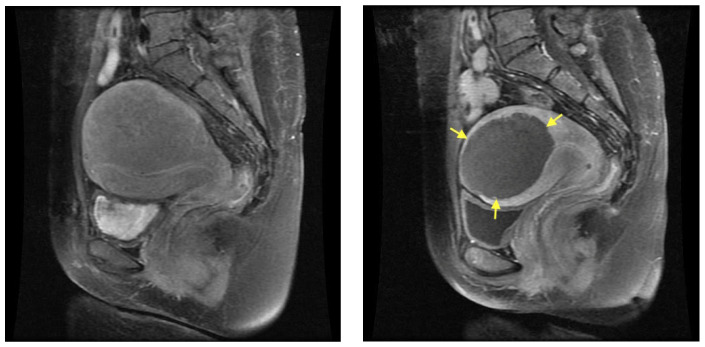
Magnetic resonance (MR) images of adenomyosis from a 47-year-old woman: (**Left**) pre-HIFU MR image showing extensive adenomyosis involving mostly the posterior fundal uterine wall; (**Right**) post-HIFU MR image, 6 months after treatment, showing a hypoperfused well-defined area (arrows) as the result of HIFU.

The median baseline volumes of the uteri and the measurable adenomyotic lesions were 446.6 cm^3^ (range: 240–1488.6 cm^3^) and 97.7 cm^3^ (range: 43.7–367.4) cm^3^, respectively. Table 3 summarizes the same volumes at 6 and 12 months after treatment, as well as the percentage volume reduction. Five patients (33.3%) had adenomyosis in the anterior uterine wall, nine (60.0%) in the posterior wall, and one (6.7%) in both the anterior and posterior walls. The images of one of the patients before and 6 months after HIFU are shown in Figure 3.

The median menstrual pain scores (MPS) and the modified UFS-QOL scores before treatment were 5.5 (range: 3–8) and 30 (range: 21–33), respectively. The scores at 6 and 12 months after treatment are summarized in Table 3.

The longest duration of follow-up was 62 months. Six (40.0%) patients had attended follow-up for more than 2 years. Ten women (71.4%) had completed follow-up or had been referred out to primary care, and four women (28.6%) are still attending ongoing follow-ups. Five patients (35.7%) required additional intervention after HIFU treatment. Three patients had hysterectomy 17, 32, and 50 months after HIFU due to persistent heavy menstrual bleeding. One of them had the largest uterus volume (1488.6 cm^3^) in this series, but she strongly preferred uterus preservation. One patient had an adenomyomectomy in another center, and one patient had repeat HIFU 10 and 15 months after their initial treatment, respectively.

Two patients had major complications. The occurrence of complications is listed in Table 1. One patient had a thermal bowel injury needing small bowel resection, which had been reported previously and was suspected to be due to overly extensive ablation of the adenomyotic lesion (SIR Class D) [15]. The other patient had prolonged nerve injury with buttock pain and bilateral lower limb weakness, which completely recovered after 6 months of physiotherapy and walking support (SIR Class C). None of the patients had become menopausal.

One woman had successful spontaneous pregnancy 24 months after HIFU. She had an unremarkable antenatal course and had emergency cesarean delivery for failed induction at 40 weeks’ gestation. The baby weighed 3135 g, with Apgar scores of 9 at 1 min and 10 at 5 min.

## 4. Discussion

HIFU employs focused ultrasound waves to generate heat and induce thermocoagulation necrosis at a specific target without damaging the adjacent tissues. It aims to deliver heat of over 60 °C at the target tissue [6,7,8,16]. Treatment for tumors at the liver, pancreas, kidney, and uterus was enabled by its unique ability [16]. The HIFU beam can be guided under MR or ultrasound imaging for target localization. HIFU uses grey-scale changes to determine the adequacy of tissue ablation and is generally considered cheaper and requires shorter treatment time than MR-guided HIFU.

In Hong Kong, Leung et al. has demonstrated that HIFU is effective in treating symptomatic uterine fibroids [17]. Symptom scores of fibroid-related abdominal pains, the pictorial chart, and Incontinence Impact Questionnaire scores were significantly improved. Median volume shrinkage at 3 months was 17.2% (95% confidence interval: 4.3–26.6%). A modified treatment protocol with oxytocin augmentation showed promising results compared to control HIFU with standard protocol with regard to fibroid volume reduction and symptoms relief [18]. Our preliminary study, which was reported in 2019, showed a substantial improvement by 44.9% in symptom severity score and a reduction in fibroid volume of 75.9% when compared to pre-treatment at 12 months post-treatment [10]. Our current study shows similar findings with a reduction in fibroid volume of 66.9% (−33.7–98.3%) and significant symptom relief of 50% (0–73%) at 12 months. Furthermore, our study also demonstrates favorable long-term outcomes of patients who have received HIFU ablation with sustained symptom relief after treatment.

The efficacy of HIFU in treating adenomyosis is less well established. Based on our study, it was found that the median uterine volume reduction was 41.9% at 12 months following HIFU therapy, with values ranging from 18.9% to 69.2%. It was also shown that symptoms such as dysmenorrhea and menorrhagia were decreased, as reflected by improvements of the MPS and SSS. This is consistent with other studies reporting a considerable degree of uterine volume reduction and symptoms improvement after HIFU [19,20,21,22,23].

Treatment success is ensured by strict patient selection. Patients who have substantial pelvic adhesions from previous laparotomies or severe pelvic endometriosis are typically contraindicated for treatment as they are at a higher risk of complications [7]. In our cohort, there was a patient with bowel injury diagnosed 8 days after treatment, which was suspected to be due to extensive ablation [15]. She suffered from posterior wall adenomyosis, which was technically more demanding owing to the distance between the transducer and target lesion. The risk of intestinal injury could be mitigated through thorough pre-operative bowel preparations and carefully controlled ablation range [15]. Perhaps more detailed pre-operative assessments of factors such as pelvic adhesions or endometriosis, which may alter treatment outcomes and complication risk, can be performed to include a cost–benefit analysis of this technique compared to other treatment options.

When compared with other treatment modalities, HIFU is known to have more re-interventions [24,25] but comparable treatment benefits [26,27]. Our adjusted re-intervention rate was 10.8% when hysteroscopy myomectomy was considered a combination treatment with HIFU ablation for uterine preservation [28]. For patients with adenomyosis, the re-intervention rate was 35.7% and illustrates that HIFU could potentially act as an adjunct to other therapies. This is particularly useful in high-risk patients with poor pre-morbid status, such as our patient with Eisenmenger syndrome [29] and another one with deep vein thrombosis, when conventional treatment, including prolonged operations under general anesthesia or the use of antifibrinolytics, would not be ideal. Further prospective studies could explore responses after HIFU and combination therapy, as HIFU combined with gonadotrophin-releasing hormone agonist (GnRH-a) and levonorgestrel-releasing intrauterine system (LNG-IUS) have been shown to provide better outcomes [30].

Our study has some limitations due to its retrospective design and the relatively small number of patients. Patients may have re-interventions in other centers or countries without our knowledge. The lack of data on post-treatment non-perfused volume in our series makes comparison with other similar studies difficult. Despite these drawbacks, HIFU represents a potential development in the treatment of fibroids and adenomyosis, and clinicians will be able to give their patients the best treatment alternatives for these conditions.

## 5. Conclusions

HIFU offers a uterus-sparing alternative for patients with uterine fibroids and adenomyosis. Our results add to the existing literature and show that HIFU is safe and effective in local settings. Even though HIFU is still considered relatively novel in some countries, it is gaining popularity both locally and internationally because of its encouraging efficacy and safety data and quick recovery time. With stringent patient selection and planning, HIFU can also be used as an adjunct to other treatment options, such as bridging to hysteroscopic myomectomy or delaying hysterectomy as the definitive treatment. Our data provide reassurance to clinicians that HIFU can be a useful treatment alternative for fibroids and adenomyosis.

## Figures and Tables

**Table 1:** Patient characteristics, treatment details, and outcomes.

	Fibroids	Adenomyosis
Characteristics		
No. of patients	65	14
No. of treatments	66	15
Age (years, range)	45 (36–53)	46 (37–50)
Treatment details		
Treatment time * (minute, range)	123.5 (46–208)	99 (62–178)
Sonication time † (second, range)	1508 (541–2445)	1218 (419–2006)
Energy delivered (joules, range)	587,503 (207,303–959,971)	354,848 (111,897–771,356)
Follow-up		
Duration (months, range)	26 (3–95)	22 (12–62)
Re-intervention		
Overall rate	21.50%	35.70%
Hysterectomy	*n* = 5	*n* = 3
Myomectomy (vaginal/hysteroscopic)	*n* = 7	0
Adenomyomectomy	0	*n* = 1
Repeat HIFU	0	*n* = 1
Levonorgestrel intrauterine system insertion	*n* = 2	
Adjusted rate ‡	10.80%	NA
Complications		
Major (SIR Class C–F): rate	1.50%	13.30%
Pelvic inflammatory disease and septicaemia	*n* = 1	0
Thermal bowel injury	0	*n* = 1
Prolonged pain due to nerve injury	0	*n* = 1
Minor (SIR Class A–B): rate	18.20%	13.30%
Second-degree skin burn	*n* = 2	*n* = 1
Urinary tract infection	*n* = 2	*n* = 1
Pelvic, back or leg pain	*n* = 8	0

NA: not applicable. Data are given as median (range), unless specified. * Treatment time = time from the first to the last sonification. † Sonification time = time of ablation when energy was being delivered to the target. ‡ Adjusted rate = calculated after exclusion of vaginal/hysteroscopic myomectomies.

**Table 2:** Fibroid volume and symptom severity scores (SSS) after HIFU.

	Pre-Treatment	6 Months	12 Months
Fibroid volume (cm^3^, range)	190.1 (18.5–1729.4; *n* = 75)	91.2 (2.4–1511.0; *n* = 73)	59.6 (1.4–1052.3; *n* = 59)
Volume reduction (%, range)	NA	50.1 (−26.2–97.8)	66.9 (−33.7–98.3)
*p* *	NA	<0.00001	<0.00001
SSS (range)	28 (19–35)	16 (8–22)	14 (8–23)
SSS reduction (%, range)	NA	44 (0–70.3)	50 (0–73)
*p* *	NA	<0.00001	<0.00001

Data are given as median (range). * Compared to pre-HIFU. NA: not applicable; SSS: symptom severity score.

**Table 3:** Uterus and adenomyosis volume and MPS after HIFU.

	Pre-Treatment	6 Months	12 Months
Uterus volume (cm^3^, range)	446.6 (240–1488.6; *n* = 15)	431.8 (166.8–1170.9; *n* = 15)	247.9 (150.3–687.26; *n* = 11)
Uterus volume reduction (median %, range) +	NA	19.6 (1.2–42.0)	41.9 (18.9–69.2)
*p* *	NA	0.28014	0.04136
Adenomyosis volume (cm^3^, range)	97.7 (43.7–367.4; *n* = 13)	66.1 (17.2–249.4; *n* = 13)	69.9 (11.1–202.8; *n* = 10)
Adenomyosis volume reduction (%, range) +	NA	25.5 (2.1–78.4)	44.5 (−20.9–96.2)
*p* *	NA	0.11184	0.07508
MPS (range)	5.5 (3–8)	2(0–5.5)	3(0–6)
MPS reduction (%, range)	NA	60 (−83.3–100)	50 (−100–100)
*p* *	NA	<0.0001	0.0004
SSS (range)	30 (21–33)	16 (10–28)	16 (9–28)
SSS reduction (%, range)	NA	38.1 (6.6–66.7)	40 (0–70)
*p* *	NA	<0.0001	<0.0001

Data are given as median (range). * Compared to pre-treatment. NA: not applicable; MPS: menstrual pain score; SSS: symptom severity score. + % volume reduction is given as median of all % volume reduction from the data set.

## Data Availability

The raw data supporting the conclusions of this article will be made available by the authors on request.
